# Canadian research ethics board members’ attitudes toward benefits from clinical trials

**DOI:** 10.1186/s12910-015-0079-8

**Published:** 2015-12-02

**Authors:** Kori Cook, Jeremy Snyder, John Calvert

**Affiliations:** Simon Fraser University (Canada), 8888 University Drive, Burnaby, BC V5A 1S6 Canada

**Keywords:** Post-trial access, Fair benefits, Reasonable availability

## Abstract

**Background:**

While ethicists have for many years called for human subject trial participants and, in some cases, local community members to benefit from participation in pharmaceutical and other intervention-based therapies, little is known about how these discussions are impacting the practice of research ethics boards (REBs) that grant ethical approval to many of these studies.

**Methods:**

Telephone interviews were conducted with 23 REB members from across Canada, a major funder country for human subject research internationally. All interviews were digitally recorded and transcribed verbatim. After coding, the data was analyzed to identify central themes and topics. Themes were identified, application of the themes was confirmed, and these themes were then used to populate the findings of this manuscript.

**Results:**

Our analysis of the interviews identified two primary themes when considering what benefits are owed to research participants and their communities. 1) Most study participants felt that given that these studies are led by persons in the role of researcher rather than health care provider, they had a limited obligation to provide benefits to study participants. 2) These REB members were all working in Canada, a high income country where most residents enjoy high levels of access to health care. As a result of this context, the study participants tended to focus on ethical concerns including obtaining informed consent and avoiding undue inducement to participate in research rather than ensuring that study participants directly benefit from successful trials.

**Conclusions:**

Research on REB members’ attitudes toward what benefits are owed to study participants and community members is needed in other countries in order to determine how context affects these attitudes.

## Background

Before a medical intervention can be judged effective it must generally be evaluated through clinical trials, which hinge on the participation of human subjects. While there are strict requirements relating to the treatment of human participants during clinical trials, there are often few requirements around a researcher’s obligation to participants once the trial ends [1]. In the case of pharmaceuticals and other intervention-based therapies, this could mean access to a potentially beneficial treatment would end when the trial ends.

Benefits accruing from trial participation, including post-trial access to the pharmaceutical, therapy, or intervention under investigation after the study period, can be overlooked as an ethical issue for researchers [2, 3]. While post-trial access may not be such a pressing issue for participants in high-income countries with access to publically funded drug plans, participants in other parts of the world may lose access to beneficial treatments at the end of a trial [4]. A similar and related concern exists around benefits for community members, particularly in low-income communities and countries. Community members may shoulder some of the burdens of research trials, such as supporting friends and family or being affected by new infrastructure, even if they do not participate directly in the research or gain any direct benefits from the trial [5, 6].

Research Ethics Boards (REBs) are a key group in evaluating the ethical requirements of human subject research, but little is known about their views towards benefits for participants and other community members. One of the first attempts to investigate the views of ethics board members was by Pace et al. (2006) who used telephone and self-administered surveys to study attitudes towards post-trial access. Pace reported contextual difference between REB members’ views, with chairs from Europe and South America more likely than those in the United States, Australia, and China to endorse providing drugs from the trial free of charge to participants [7]. More recently, Dainesi and Golbaum (2012) used an e-survey to investigate the views of major stakeholders in Brazil around post-trial access, including clinical investigators and ethics committee members. The majority of ethics committee members questioned believed that all participants should have access to the studied intervention post-trial [8].

This paper builds on this work by examining the attitudes of REB members regarding what benefits are owed to research participants and their communities and the ethical rationale for requiring these benefits. The 23 REB members interviewed in this study are all based in Canada, a high-income country that is and will continue to be a funder country for clinical trials in low-income countries and therefore serve as an initial gatekeeper via research ethics review of studies that would take place amongst vulnerable populations. These REBs are governed by the revised Tri-Council Policy Statement (TCPS2) on Ethical Conduct for Research Involving Humans, which states that prior to conducting research with human participants, researchers should “outline the scope and nature of potential benefits that may accrue to participants during and after he research” and “ensure that the proposed distribution of benefits is fair, without imposing undue burdens on the researcher that would make it too difficult or costly to complete research” [9]. Thus, while TCPS2 notes that fairness in the distribution of benefits is a concern for researchers, it does not provide clear guidance on what benefits from clinical trials are necessary, making better understanding of the attitudes of Canadian REB members on this issue of great value.

## Methods

The purpose of this qualitative study was to explore attitudes of REB members regarding benefits owed to research participants and communities. To address this, phone interviews with REB members were conducted in the spring of 2014.

### Ethics, consent, and permissions

Participant recruitment started after approval for this study was received from the Research Ethics Board at Simon Fraser University. Using only publicly accessible information on university websites, REB members were contacted via email. The email contained information about the study, contact details of the researchers, and offered a $50 honorarium. Consent to participate was received from all participants.

### Recruitment

The researchers identified all Canadian universities with associated medical schools, and used public information on the universities’ websites to identify REB members where this information was publicly available. These membership lists included academic and lay members who constituted the REB. All participants were current or recent REB members. Where information was available, the researchers contacted members of REBs that reviewed research involving biomedical, health sciences, and health social sciences. Snowball sampling was not used as those universities that published REB membership lists published complete lists and REB members were not expected to have knowledge of REB membership at other universities.

REB members were recruited from Alberta (*n* = 1), British Columbia (*n* = 7), Manitoba (*n* = 1), Ontario (*n* = 11), Saskatchewan (*n* = 4). They represented a range of academic disciplines, including medicine (*n* = 6), ethics (*n* = 2), kinesiology (*n* = 2), nursing (*n* = 2), pharmacology (*n* = 2), psychiatry (*n* = 2), engineering (*n* = 1), environmental studies (*n* = 1), geography (*n* = 1), health sciences (*n* = 1), linguistics (n = 1), plant sciences (*n* = 1), and social sciences (*n* = 1). No lay REB members contacted agreed to participate in this study.

### Data collection

In total, 23 phone interviews were completed, each lasting approximately 45 minutes and conducted by one researcher. Participants had varying experiences with the subject matter being discussed in the interviews, and had varying research and academic backgrounds, resulting in a wide variety of views on the subject matter.

As the researchers were trying to capture a broad array of views on the subject matter, certain terms used in the interviews were kept intentionally general. “Research participants” were defined as those directly participating in research, while “community members” were defined as those not directly participating in research, but who may be impacted by the research in some way. “Benefits” could be any positive impact of research, be it tangible or intangible. Tables 1, 2, 3, 4 and 5 contain sample questions asked of the research participants, each of which was followed by more specific probes.

**Table. 1:** Are there requirements around benefits for research participants?

Response	Count
No	12
No direct benefit, but a societal benefit	7
Proportional benefit	3
Unsure	1

**Table 2:** What do you think is morally required, in terms of benefits for participants?

Response	Count
Knowledge	13
Not worse off, compensated for expenses or time	11
Continued access to interventions	6
Whatever benefits were specified in the consent form	5
No benefits	1

**Table 3:** Why are these benefits for participants morally required?

Response	Count
Transparency	5
Offering anything would be coercive	3
Cost prevents offering benefits	2
Not the role of the researcher	1
Fairness and reciprocity	14

**Table 4:** Are there requirements around benefits for community members?

Response	Count
Nothing	9
Generalizable knowledge	8
Study results	2
Unsure	1

**Table 5:** What do you think is morally required, in terms of benefits for participants?

Response	Count
Access to information	18
Access to the intervention	3
No benefits	2

### Analysis

All interviews were digitally recorded and transcribed verbatim. The transcripts were uploaded into NVivo qualitative data management software and reviewed by the first and second authors. A coding scheme was created and applied to the transcripts in NVivo following the first two co-authors conducting an independent review of the transcripts. The first two co-authors met to compare observations on these transcripts and reached consensus on a set of dominant issues emerging from the transcripts that would be used for developing the coding scheme. The transcripts were coded by the first author and confirmed by the second author in order to support the credibility of the application of the coding scheme. After coding, the coded data was analyzed by the first two co-authors to identify central themes and topics. Themes were identified, application of the themes was confirmed by the first and second authors who also confirmed representativeness of the themes across transcripts, and these themes were then used to populate the findings of this manuscript.

## Results

### Benefits for participants

Respondents indicated that they were familiar with the challenges and issues around providing benefits for participants, and most explained that they had considered these issues through their role as REB members. Most respondents indicated that Canadian law does not require benefits for research participants (see Table 1), although there was some confusion around “legal” requirements following from TCPS2 versus common practices. Several respondents specified that their role as an REB member was to ensure protection from harm, but that this did not involve ensuring benefits for participants. As one respondent explained, *“The ultimate goal of an ethics committee is to maximize the safety of a person who volunteers to participate in a study. There is absolutely no guarantee of benefit and that’s supposed to be stated on virtually all consent forms.”* Another common concern was that while no benefits are required, it is crucially important that researchers clearly communicate this to participants during the consent process.

There was some confusion around what was explicitly required by the REBs and what was common practice. As one respondent noted, *“I think the requirements are a little fuzzy… Rather than being direct in stating what is expected or what is required. I think that speaks to the different funding arrangements and the different policy frameworks…”* Over the course of the interviews, several participants singled out funders as having some control over benefits for participants, or dictating benefits in some way.

A key recurring theme throughout almost all of the interviews was a concern around undue inducement to participate in a study. One REB member stated, *“…it’s pretty unclear as to what …is defined as an appropriate amount of compensation for folks, when does something become more coercive than others.”* Approximately one third of respondents took a broad view of benefits for participants and indicated that while no direct benefits were required by REBs, all research had to have some expected benefit for society as a whole such as contributing to new advances in technology or to societal knowledge in general. One respondent explained, *“you can’t just go around sticking pins into people and drawing their blood unless there’s at least a legitimate intent to … provide a cure for something or to understand a disease.”*

A few respondents specified that the potential benefits to participants be proportional to the risk they experienced through their participation in the research, with some respondents indicating that this is often a difficult balance to strike: *“My understanding is that we’re always looking at proportionality, …that there is some kind of balance for that in terms of what the person will get, either directly or indirectly.”*

When respondents were asked what they themselves thought was morally required in terms of benefits to participants, regardless of existing legal requirements or norms, the answers were markedly different from those around legal requirements. The two most common responses had to do with compensating participants for their time and expenses and for increasing knowledge (see Table 2). Compensation for participants’ expenses included reimbursing them for transportation costs, as well as providing financial compensation for their time. Several participants made a clear distinction between “benefits” for participants and “compensation” for time and expenses: *“definitely the out of pocket expenses, I believe is a bare minimum.”* Others considered compensation to be an impermissible benefit.

Again, undue inducement was mentioned several times as a key consideration when compensating participants. One researcher described this dilemma in the context of their own research: *“…what I offer to do in my own research is pay any sort of transit or transportation costs and then I have a small honorarium that sort of represents the hour that they’ve given for an interview … But I think that’s very different than paying someone $500 to do an interview.”*

Increasing knowledge was the second common theme around morally required benefits for participants. For some, this meant a commitment to research that will further our collective understanding of a disease or therapy, while others included self-knowledge and understanding gained through the research process. Multiple respondents described access to study results as the “minimum” for ethical research. For one respondent, increasing knowledge comprised several areas: *“…I think they need to learn something from the process… I think they need to learn their own medical results and… I think that they have a right to know what the trial accomplished.”*

Several respondents focused not on the specific benefits that participants should receive, but on the transparency of the consent process. These respondents felt that participants are entitled to whatever was specified in the consent form, and stressed that true consent from participants was more important than the precise benefits offered. Generally, these responses emphasized the need for informed consent and the ability of participants to choose to participate without undue inducement or coercion. One researcher described this view as being “*a pretty typical one*,” while another explained, *“… the requirement is really to make it clear what benefits are including, including in many cases none.”*

A final recurring theme was continued access to an intervention or therapy as a moral requirement of research. Some felt that this should be a requirement for all research, while others specified that access to the intervention being studied should only be extended past the end of the study if no other alternatives are available: *“…if it’s truly a unique medication for a unique problem then there should be some way in which they can get privileged access.”* Several respondents indicated that while they supported continued access to an intervention, they had reservations about the practical implementation of such a requirement: *“…if you’re going to say … these benefits have to keep going after the research I think there’s going to be a lot of research …that will stop because of the money issue.”*

When asked *why* they believe certain benefits are morally required, some participants provided rationales for *not* providing benefits, such as: it is not the role of researchers to offer benefits; cost prevents researchers from offering benefits; offering anything would be coercive; and transparency, including honouring consent forms (see Table 3). One respondent explained that researchers are *“not out there to continue to treat, they’re actually there to do the research and provide the knowledge,”* and that *“it’s up to the… country and the provinces to decide what they want to actually provide.”* Another respondent explained that, *“funding is difficult to come by”* and requiring certain benefits would limit unfunded research conducted by students and professors. Several respondents believed that providing any benefits at all could be coercive and unethical. These respondents stressed the “*voluntary*” and “*non-obligatory*” aspects of research participation. Again, several respondents stressed that as long as participants freely agree to participate in research, no additional benefits are morally required.

The rationales for providing benefits focused almost exclusively on fairness and reciprocity. Many respondents cited proportionality as a consideration within fairness, with one respondent explaining, *“… it’s definitely ethically desirable that participants receive some benefit from participating in a trial that is related to the risks that they’re assuming and the time that they’re putting in as research subjects.”* Other respondents emphasized knowledge exchange and a “*quid pro quo*” approach to research.

### Benefits for community members

The interview participants generally struggled with specifying benefits for community members, with several participants explaining that they had never considered benefits for community members in their role as an REB member. The most common responses were that community members were either owed nothing or were entitled to generalizable knowledge gained through the study (see Table 4). One respondent cited a *“vague notion that the research is good for everybody”* while another explained that research *“has to have some kind of outcome that the larger society could draw on and build… on.”* Several respondents also stressed the need for communicating study results as *“an obligation to make your findings public.”*

Respondents were asked what benefits they believed community members should receive, regardless of existing REB requirements or legislation. These responses fell into three categories: no benefits; access to information about the results (including broader societal impacts of the information generated from the study such as new technologies or polices); and access to the intervention being researched (see Table 5). Well over half of respondents cited access to information generated by the study as a requirement for conducting ethical research, but benefitting from the knowledge gained through research clearly varied in definition between respondents. Some felt this was limited to having access to the final study results, while others believed that this knowledge should be translated into improvements in the community, such as policy changes or improvements in health and medical treatments and procedures. One respondent voiced that researchers don’t value communication with communities and stakeholders as much a publication in peer-reviewed publications.

A few respondents felt that community members should also have access to the therapy or intervention under investigation if it demonstrated a benefit to participants. One respondent explained that as pharmaceutical companies were profiting and benefiting from tax breaks, *“…therefore the obligation should be that they are contributing to the health and well-being of the community…”* Another respondent explained, *“…ethically there absolutely is an obligation on the sponsor in this case to make efforts to ensure that the intervention is available and to contribute to addressing problems that might frustrate that intervention from being available.”*

When asked for an ethical rationale as to why community members should benefit from research, respondents generally struggled to define a specific rationale. Some respondents indicated that benefits and an ethical rationale were dependent on a specific definition of community, such as within certain geographic boundaries or defined by some common connection. Several respondents evaded the question, while again referring to vague benefits, such as knowledge exchange. Interestingly, several respondents cited an ethical obligation to taxpayers as a rationale for providing benefits to community members: *“…if Canadians are paying for research…there should be benefits for Canadian communities.”*

## Discussion

Our analysis of the interviews identified three challenges when considering whether and what benefits are owed to research participants and their communities. Many REB members understood providing access to the intervention as being the role of the state, not the role of the researcher. Similarly, many of the views on benefits were shaped by the fact that REB members were based in Canada, and expected the state to fulfill certain health obligations. The interviews also highlighted that more consideration, awareness, and research is needed around benefits for participants and communities.

### Identifying roles

The Canadian REB members we spoke to identified researchers and the state as having distinctly different roles with respect to health research and healthcare provision. Respondents generally referred to the role of researchers as being solely focused on research – not with providing healthcare. Several respondents indicated that research participants should not receive any benefits, they later went on to explain that it was extremely important that participants, community members, and citizens all have access to a beneficial intervention through the healthcare system. Those respondents who did not believe any benefits were necessary were largely still concerned with access to medicines, but did not feel that providing this access was the responsibility of researchers. While some respondents stressed the importance of access to medicines, this was often expressed as being an issue unrelated to ethical research. Similarly, most felt it is not the role of REBs to compel researchers to provide healthcare.

There was a concern from some respondents that researchers were not doing enough to provide benefits for participants, or that researchers were not even considering benefits for participants and communities in ethics applications. However, few respondents supported requiring researchers to include specific considerations around benefits in their ethics applications. This response mirrors the belief that researchers and the state have distinct roles with respect to providing access to healthcare and that research participants be clearly advised that researchers do not have a therapeutic role [10]. Several respondents expressed a desire for researchers to be more aware and proactive in choosing to provide benefits for participants, but shied away from recommending legislation or requirements forcing researchers to provide certain benefits. The rationale for this was the fact that every study is different, and there was a reluctance to mandate the same requirements for all types of research.

Several respondents made statements that indicated that researchers sponsored by pharmaceutical companies were different than the “average” researcher and that different requirements might apply to them. There was a general view that the “average” researcher is a university-based academic, professor or otherwise, and several respondents cited pharmaceutical companies as being a different case entirely, facing correspondingly different ethical requirements. Because these companies were presumed to be making substantial financial gains based on the results of the study, REB members may have felt that their obligations to participants are stronger and more stringent. This raises issues around whether who is conducting or funding the research creates special requirements around who benefits and what benefits they should receive, and also around whether or not financial gain creates special requirements and obligations around benefits. This also raises broader concerns around addressing inequalities and power imbalances in research, particularly in low-income settings where participants may be more vulnerable.

### Research Ethics Board Members' Context

The assumption that the role of the researcher is fundamentally different from that of the state in terms of access to medicines and healthcare is likely rooted in the fact that all of the researchers interviewed are based in Canada, a country with a comprehensive national healthcare system. Their context of a high-income country with a well-functioning health system likely shaped their assumptions and focus. The assumption that the state is responsible for – and will provide – access to needed medical care frees researchers to focus on research, and to trust that advances will be accessible to the general population. It’s worth noting, however, that even in the Canadian context, access to affordable pharmaceuticals is not guaranteed. According to the most recent estimate of the Canadian Institutes of Health Information, governments paid only 41.6 % of pharmaceutical expenditures in Canada in 2013 [11].

With respect to individual participants, respondents were more concerned with obtaining informed consent and freedom from undue inducement than with ensuring participants had access to the intervention being studied. For community members, there was a general sense of confusion around *why* community members should benefit, possibly due to existing community access to healthcare in Canada. The fact that “community” is a difficult concept to define and the definition was left intentionally broad in the interviews likely also contributed to the confusion.

### Limitations

Since some universities do not publish REB members’ names or their contact information on their websites, the researchers did not contact REB members from all medical schools in Canada. There was no publicly accessible information for schools in Quebec and Eastern Canada, thus these REB members from these regions are not represented in this study. As a result, experiences that are distinctive of that region and particularly norms in the predominantly French speaking regions of the country are not well represented in this study.

## Conclusion

Our discussions with REB members based in Canada highlighted assumptions around access to healthcare and the role of the researcher that may not translate to other countries and communities. In the Canadian context, investigators can focus on conducting research, while trusting, perhaps naively, that the state will provide access to beneficial health innovations. This is simply not be the reality in many low and middle income settings. The effect of local context on shaping the concerns of Canadian REB members demonstrates the importance of requiring ethical approval from the country where the trial is being conducted as well as the country conducting the investigations, as REBs in the host country will likely be more attuned to the research context in their communities. Above all, our research highlights a need for continued discussion and awareness around these issues and the need for further investigation that includes the perspectives of both communities funding human subject research and those in which this research takes place. In this sense, REB members in Canada might be seen as ‘well-meaning amateurs’ [12] who would benefit from additional education as to these ethical issues while REBs would benefit from additional professionalization.

## Abbreviations

REB: Research Ethics Board
TCPS2: Revised Tri-Council Policy Statement
